# Electron Transfer between Electrically Conductive Minerals and Quinones

**DOI:** 10.3389/fchem.2017.00049

**Published:** 2017-07-13

**Authors:** Olga Taran

**Affiliations:** Department of Chemistry, Emory University Atlanta, GA, United States

**Keywords:** electrochemistry of minerals, electrochemistry of quinones, iron sulfides, geobattery, pyrite, redox gradients, origin of life

## Abstract

Long-distance electron transfer in marine environments couples physically separated redox half-reactions, impacting biogeochemical cycles of iron, sulfur and carbon. Bacterial bio-electrochemical systems that facilitate electron transfer via conductive filaments or across man-made electrodes are well-known, but the impact of abiotic currents across naturally occurring conductive and semiconductive minerals is poorly understood. In this paper I use cyclic voltammetry to explore electron transfer between electrodes made of common iron minerals (magnetite, hematite, pyrite, pyrrhotite, mackinawite, and greigite), and hydroquinones—a class of organic molecules found in carbon-rich sediments. Of all tested minerals, only pyrite and magnetite showed an increase in electric current in the presence of organic molecules, with pyrite showing excellent electrocatalytic performance. Pyrite electrodes performed better than commercially available glassy carbon electrodes and showed higher peak currents, lower overpotential values and a smaller separation between oxidation and reduction peaks for each tested quinone. Hydroquinone oxidation on pyrite surfaces was reversible, diffusion controlled, and stable over a large number of potential cycles. Given the ubiquity of both pyrite and quinones, abiotic electron transfer between minerals and organic molecules is likely widespread in Nature and may contribute to several different phenomena, including anaerobic respiration of a wide variety of microorganisms in temporally anoxic zones or in the proximity of hydrothermal vent chimneys, as well as quinone cycling and the propagation of anoxic zones in organic rich waters. Finally, interactions between pyrite and quinones make use of electrochemical gradients that have been suggested as an important source of energy for the origins of life on Earth. Ubiquinones and iron sulfide clusters are common redox cofactors found in electron transport chains across all domains of life and interactions between quinones and pyrite might have been an early analog of these ubiquitous systems.

## Introduction

Sources of electrochemical energy are widespread in Nature, including hydrothermal gradients, corrosion of metal-rich rocks and minerals, concentration gradients of redox active species, and streaming currents caused by the flow of electrolyte solutions thorough porous media. Only recently, however, we began to realize that this energy is also widely used by Nature in both biological and abiotic processes (Nakamura et al., 2010b; Revil et al., 2010) and that electrochemical redox reactions are important contributors to biogeochemical cycles (Nielsen and Risgaard-Petersen, 2015). The passage of electric currents through conductive minerals has been long-appreciated (Wells, 1914), and has gained a renewed interest due to its relevance to the biochemistry of hydrothermal vent systems and the origin of life (Karato and Wang, 2013; Malvankar et al., 2014; Yamamoto et al., 2017). Most of the redox processes across electrochemical gradients were previously associated with bacterial activity (Müller et al., 2016; Malkin et al., 2017) or human-made devices (Du et al., 2007). A detailed understanding of the extent and mechanisms of abiotic electron flows can complement our understanding of natural electrochemical systems and will be important for more accurate modeling of global biogeochemical cycles.

Many oxide and sulfide minerals are electrical conductors or semiconductors, with resistivity varying from 0.1 to 10^5^ Ω × m (Karato and Wang, 2013), capable of conducting electrons over centimeter-long distances between environments of different redox potentials (Nakamura et al., 2010a; Nielsen et al., 2010). Conceivably, electrical currents transported through conductive minerals could couple chemical and biochemical reactions in physically separated environments, form natural “fuel cells” and provide the energy necessary to maintain rich biological communities on the border of oxic and anoxic zones (Jelen et al., 2016).

The efficiency of electrochemical reactions depends not only on the magnitude of an applied redox potential, but also on the properties of the electrode. In general, a good electrode must be electrically conductive, chemically stable under selected conditions and provide a catalytically active surface for the reagents. There is a growing interest in using sustainable mineral electrodes for chemical reactions (Konkena et al., 2016) and a growing awareness of the contribution of electrochemistry to natural processes (Nielsen and Risgaard-Petersen, 2015). This study was restricted to electrochemistry of common iron oxides and sulfides assuming that due to their abundance they will dominate electron flow across different geochemical settings. Iron sulfides have been used as electrodes for electrochemical CO_2_ reduction in systems that simulate chemistry of hydrothermal vents related to the origin of life (Herschy et al., 2014; Yamaguchi et al., 2014; Roldan et al., 2015), while iron oxides have been extensively studied as terminal electron acceptors for bacterial respiration (Orsetti et al., 2013).

While filamentous and cable bacteria use electrochemical energy directly (Aklujkar et al., 2009; Seitaj et al., 2015), a wide range of bacteria are known to exchange electrons indirectly, using external electron mediators such as water-soluble flavines (Marsili et al., 2008), quinones (Newman and Kolter, 2000; Qiao et al., 2008) or, quinone-rich humic acid substances (Cervantes et al., 2000; Klüpfel et al., 2014). The addition of external quinones to microbial communities modifies interactions between microbes (Scheller et al., 2016) and changes the mode of respiration in bacteria (Cervantes et al., 2000). Interactions between organic mediators and conductive minerals may contribute to the overall flow of energy that defines biogeochemical cycles, being especially important in the environments where oxygen and solar radiation are absent (D'Hondt et al., 2004; Borch et al., 2010).

**Figure 1:**
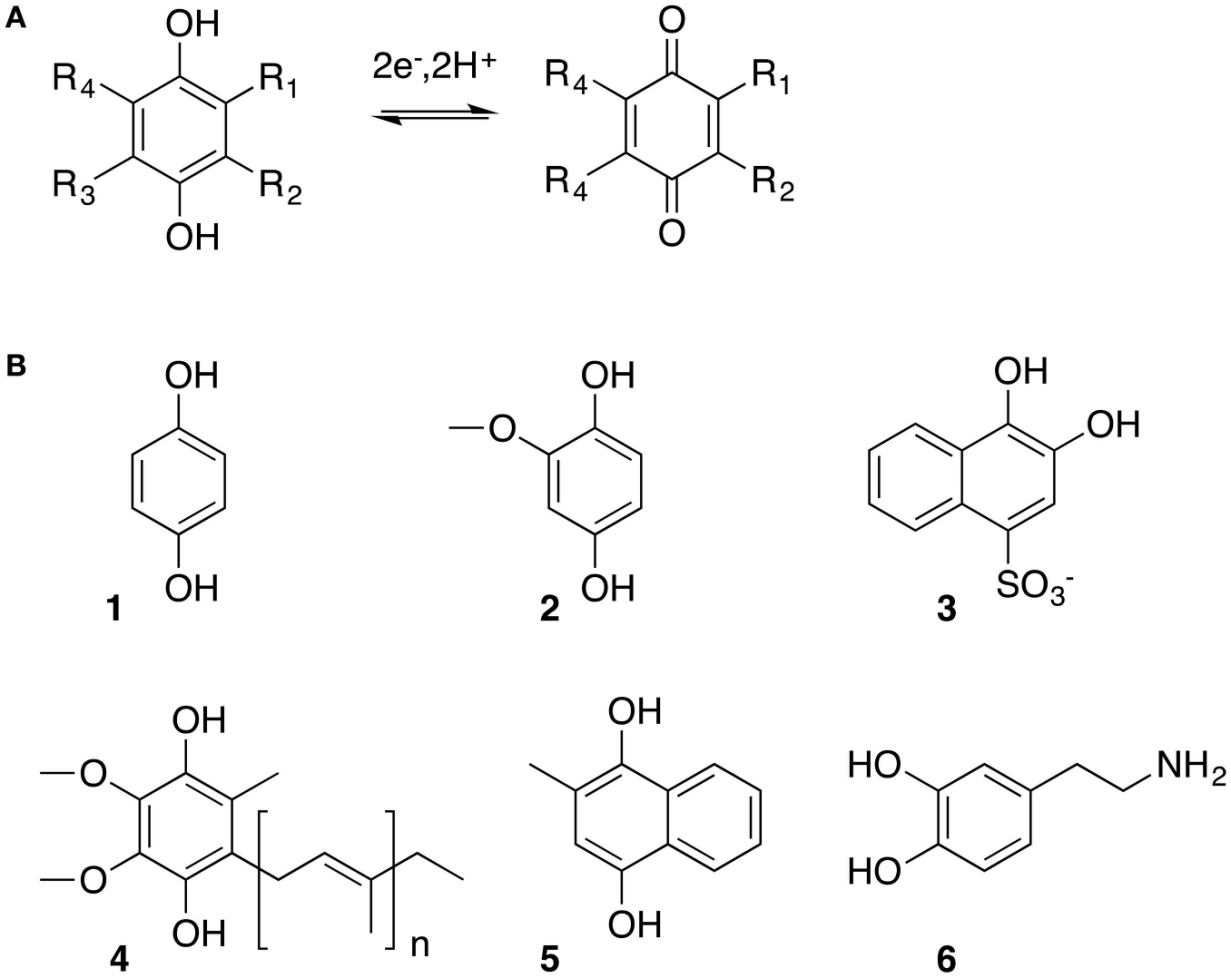
Quinone molecules are important universal electrochemical mediators: **(A)** Reversible two electron two proton redox reaction between hydroquinone (reduced) and benzoquinone (oxidized) forms; **(B)** Example of naturally occurring quinones : **1**, p-benzoquinone (BQ), simplest quinone molecule; **2**, methoxy-p-benzoquinone (MBQ), component of humic acid mixtures; **3**, 1,2-naphtohydroxyquinone-4-sulfonate (NQ), oxidation product of polyaromatic hydrocarbons, pollutant; **4**, ubiquinone, a redox cofactor, part of electron transfer chain in living cells; **5**, menadione, extracellular mediator used in bacterial respiration; **6**, dopamine, neurotransmitter.

As model organic molecules, I selected quinones (Q) and their reduced form, hydroquinones (H_2_Q), which are key electron mediators across all domains of life (Soballe and Poole, 1999) (Figure 1). Quinones are found everywhere from interstellar clouds (Bernstein, 2006) to environmental pollutants (Mousset et al., 2016). Selection of the quinones for the study broadly represented different environmental roles that these molecules have: **1** is the simplest known hydroquinone molecule and often used for model studies of quinone-mineral interactions (Uchimiya and Stone, 2006; Klüpfel et al., 2014), **2** is 2-methoxyhydroquinone, a common component on non-soluble organic matter and a plant messenger molecule (Lynn and Chang, 1990; Yuan et al., 2016), while **3** is an oxidation product of polycyclic aromatic hydrocarbons and is both a common pollutant and a likely component of prebiotic mixtures of organic molecules delivered by comets and meteorites to early Earth (Chyba and Sagan, 1992; Bernstein et al., 1999).

Electrochemical techniques are often used to study the participation of organic molecules in redox processes of redox active clays and minerals (O'Loughlin, 2008; Sander et al., 2015). Here cyclic voltammetry (CV) was used to detect hydroquinone oxidation on the surface of electrodes made from common iron oxides and sulfides. CV is an analytical technique used to monitor trace metals in marine sediments, where an increase in current is observed when a redox process takes place on the electrode's surface at a specific applied redox potential (Luther et al., 2008; Moore et al., 2009).

Differentiating the chemical contribution of electron redox cycling from usually observed microbial redox activity would improve our understanding of global biogeochemical carbon cycles. Electron transfer between conducive minerals and soluble organics can potentially generate out-of-equilibrium systems that impact environments dependent on the abiotic redox gradients, such as microbial communities around hydrothermal vent chimneys and in anoxic marine sediments (Hedrick and White, 1986; Unden and Bongaerts, 1997; Kim et al., 2012). Quinone redox cycling can affect distribution of redox active species in water columns (Nielsen et al., 2010; Nielsen, 2016) and natural bioremediation of organic molecules (Jiang et al., 2015). Finally, redox gradients have been long considered important energy source for the origin of life (Martin et al., 2008), and a mechanism of transferring energy stored in redox gradients into chemical reactions is an important step toward better understanding processes that may lead to life on Earth and other planets.

## Materials and methods

The reactivity between the mineral and redox species in solution depends on the amount of energy available in the system and the electrocatalytic properties of mineral surfaces. Overall, the energy available in the redox system depends on redox potential as defined by equation (1):

(1) $$\begin{array}{r} \Delta \text{G }=-\text{nEF} \end{array}$$

E is a measurable potential difference between reacting molecules, described by the Nernst equation:

(2) $$\begin{array}{r} E=E^{{}^{\circ}}+\frac{RT}{nF}ln\left(\frac{\left[Ox\right]}{\left[Red\right]}\right) \end{array}$$

Here E° is a standard redox potential of the redox reaction of interest, *n* is the number of electrons transferred in the reaction, T is the absolute temperature in K, and R and F are the gas and Faraday constants respectively. Naturally occurring sustained redox potentials vary from 0.2 V for marine sediments (Komada et al., 2004), 0.4 to 0.7 V for hydrothermal vents (Nakamura et al., 2010b; Ryckelynck et al., 2012) and up to 1.2 V for sulfide-rich mineral deposits (Kruger and Lacy, 1949). The naturally plausible range of redox potentials in aqueous systems is defined by the chemistry of iron and manganese, spreading from 0.7 V to −0.3 V [against a standard hydrogen electrode (SHE)] at pH 7 (Baas Becking et al., 1960). Here Ag/AgCl electrodes were used as a reference (+ 0.22 V redox potential compared to SHE), with the −0.5 to 0.5 V (Ag/AgCl) window used here broadly corresponding to the naturally occurring potential range available for redox reactions of quinone molecules.

### Materials

Pyrite, pyrrhotite, magnetite, and hematite minerals were received from Harvard Natural History Museum. Mackinawite and greigite were synthesized by previously described methods (Lennie et al., 1995; Dekkers et al., 2000) and characterized by XRD (See Supplementary Information). Quinones (**1–3**) were bought from Sigma-Aldrich (USA) and used without further purification.

Several electrode designs were tested for the study and the most reproducible data were obtained with powder electrodes described by Almeida and Giannetti (2002). Approximately 1 g of mineral was powdered with mortar and pestle for 10 min. Powdered minerals were washed several times with acetone and dried in airflow. A mixture of 2 g of paraffin and 1 g of carbon powder were heated to 70°C and a tip of a graphite rod with 5 mm diameter was immersed into the hot mixture and then pressed against mineral powder. The lateral part of the rod was covered with Teflon tape to avoid exposure to solution.

### Cyclic voltammetry

The fundamentals of CV can be found elsewhere (Compton et al., 2014), but briefly, if a molecule is oxidized or reduced on the surface of the electrode, a change of current is registered. Analysis of the change in electrical current as a function of an applied redox potential provides information about the mechanism of the reaction.

Three-electrode arrays consisted of a Ag/AgCl reference electrode, Pt-wire counter electrode (both from CH Instruments Inc., Austin, TX, USA) and homemade working electrodes made of different minerals (see above). All potentials were compared to the Ag/AgCl reference electrode (1M KCl solution, E + 0.22 V vs. SHE). Reactions were performed in 0.1 M phosphate buffer pH 7.0 in the presence of 0.4 M KCl as an electrolyte. Unless stated otherwise, all experiments were performed under an argon atmosphere. Sample solutions were purged with nitrogen for at least 10 min at the beginning of the measurements and measurements were performed with slow nitrogen bubbling through solution. To study the effect of oxygen, air was purged through the solution for 5 min, and measurements were performed in a vial open to the air with vigorous stirring between measurements. A BAS CV−50 W Voltammetric Analyzer (BASI, West Lafayette, IN, USA) was used to perform the measurements, with each measurement reproduced at least 3 times with 3 different electrode replicas for every mineral. Electrode stability was calculated by linear sweep voltammetry with a 5 mV/s scan rate, and initial activity screening was produced with 10 mM solution of the molecule of interest with a 100 mV/s scan rate. For the pH variation experiments, a modified Briton-Robinson buffer (citrate-phosphate-borate-glycine) was used. Reactions started at pH 2 and were titrated with concentrated solution of NaOH to pH 12, with pH measured *in situ* with Toledo potentiostat (Mettler Toledo, Columbus, OH, USA) and pH electrode (Hanna Instruments, Woonsocket, RI, USA) before each measurement.

### Mineral stability estimations

The range of thermodynamic stability of the minerals used in the discussion was calculated with HSC-7 software package (http://www.hsc-chemistry.com) using species concentrations found in modern ocean: [Fe]_T_ 5.0 × 10^−10^ M, [S]_T_ 2.8 × 10^−2^ M (Rijkenberg et al., 2014) and concentrations estimated to be found in Haedean and Archean oceans: [Fe]_T_2.0 × 10^−4^ M, [S]_T_ 2.0 × 10^−7^ M (Crowe et al., 2014). See a diagram in Supporting Information.

## Results

### Mineral electrode stability

**Figure 2:**
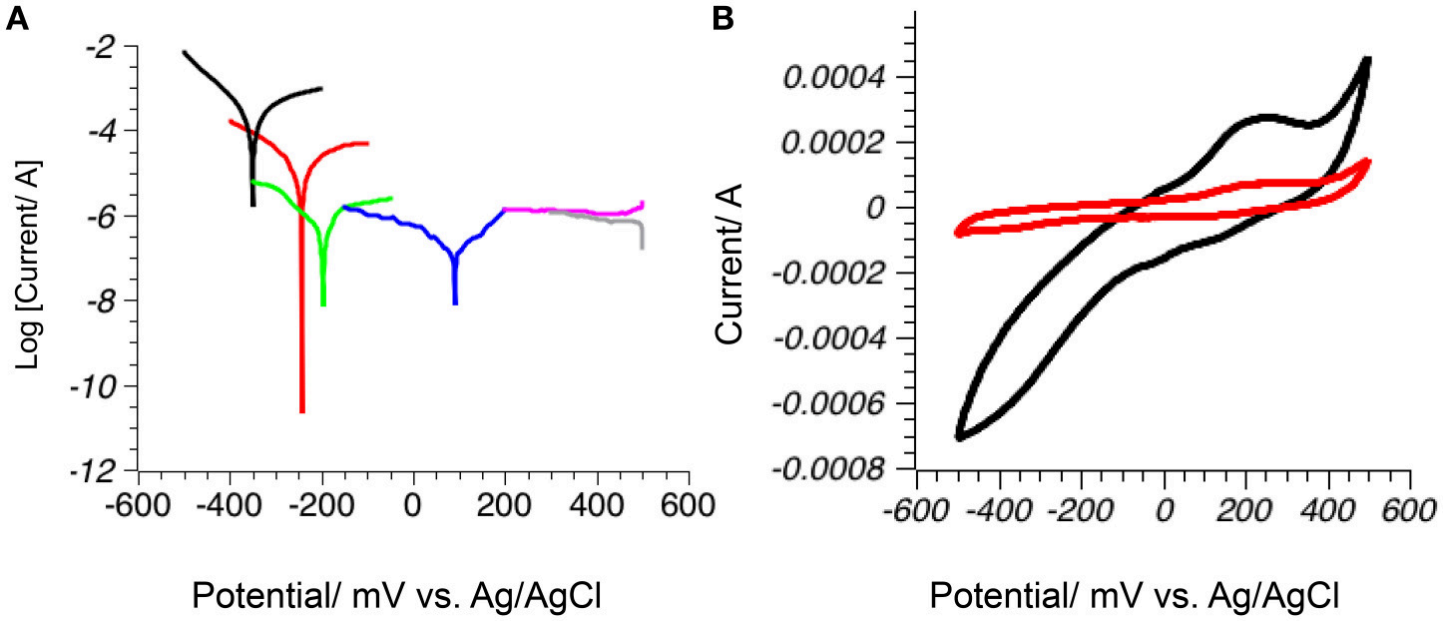
Stability of the mineral electrodes in water: **(A)** Tafel plot (log [current] vs. Potential) for mineral electrodes in phosphate buffer pH 7: mackinawite (black), greigite (red), pyrrhotite (green), pyrite (blue), magnetite (gray) and hematite (magenta); graph minimum corresponds to rest potential where oxidation and reduction reactions are at equilibrium; **(B)** Pyrite electrode is stable in anoxic atmosphere (red), but deteriorates in the presence of oxygen (black).

The stability of the selected naturally occurring minerals was evaluated by linear sweep voltammetry. The voltage applied to the electrode was slowly increased (5 mV/s) from −500 to 500 mV (vs. Ag/AgCl) and the current between the mineral and platinum working electrodes registered (Figure 2). The minimum value of each plot corresponds to the resting potential of the mineral, when oxidation and reduction processes on the surface are at equilibrium. The value on the abscissa axis is proportional to the number of electrons exchanged with solution and the overall redox stability of the sample. Minerals are reduced at more negative potentials and oxidized at more positive potentials than the minimum. In the potential range corresponding to oxidation, mineral surfaces are rich in electrons and are poor electron acceptors. Reduced metal sulfides greigite and mackinawite were less stable in solution than the iron oxides magnetite and hematite, while pyrite and pyrrhotite had intermediate stability values (Figure 2). Curves of the log |i| vs. E were asymmetric in the case of reduced iron sulfides, suggesting irreversible oxidation of these minerals. Iron oxides hematite and magnetite showed very low current values consistent with oxide dissolution inhibited in the presence of phosphate ions from the buffer (Biber et al., 1994).

When cyclic voltammetry was performed in air-saturated solutions, the presence of oxygen did not change the performance of the metal oxide electrodes (data not shown). In the case of pyrite, dramatic increases in the registered current was observed in the presence of oxygen, showing fast deterioration of the surface due to an oxygen-assisted cathodic iron dissolution pathway where oxygen oxidizes water soluble Fe(II) species into redox active Fe(III) species that catalyze the rate of pyrite oxidation (Rimstidt and Vaughan, 2003; Chandra and Gerson, 2010). This process was reversed when oxygen was removed from the system (Figure 2).

**Figure 3:**
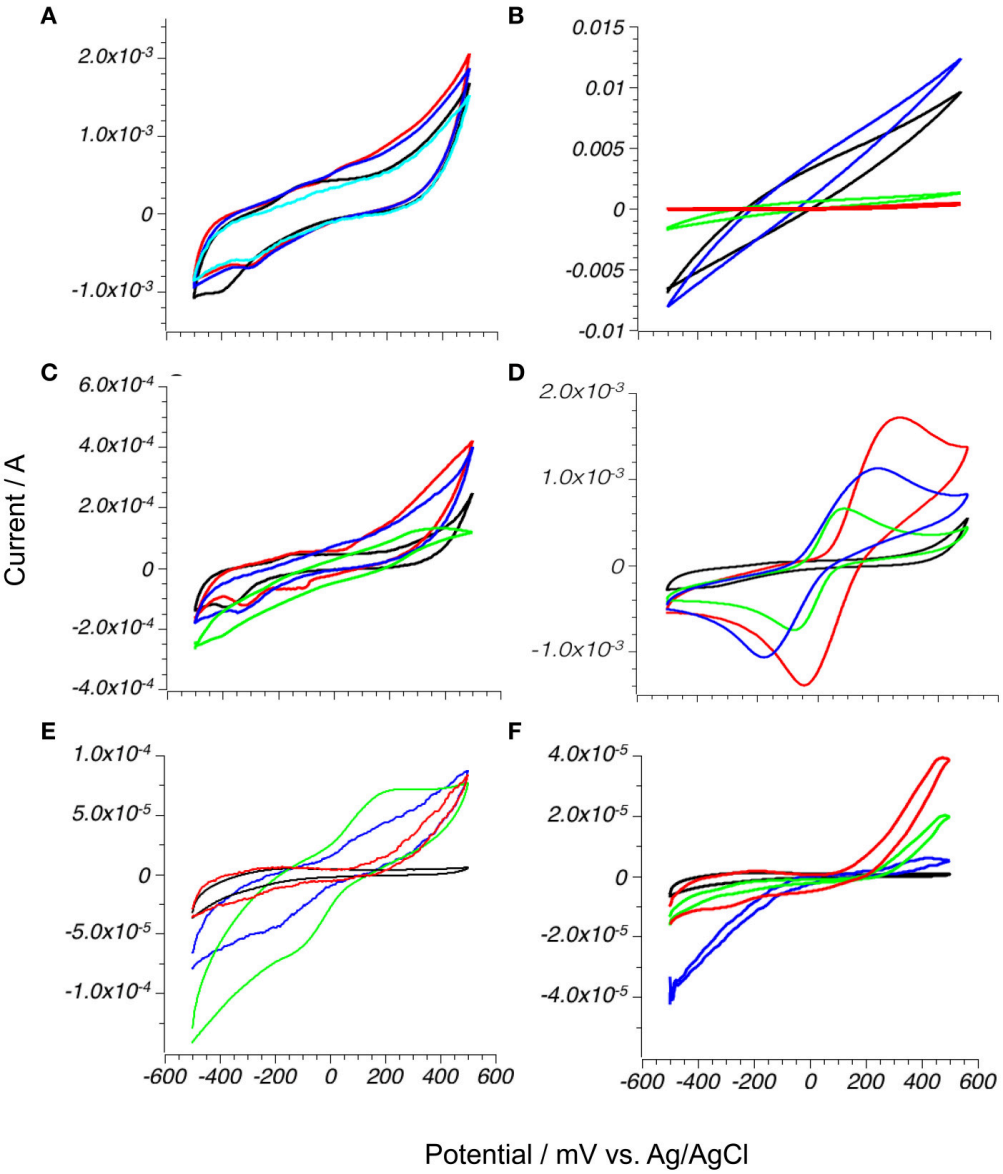
H_2_Q oxidation on mineral electrodes in anoxic environment: **1** (red), methoxy-hydroquinone **2** (blue), 1,2-hydroxynaphtoquinone-4sulfonic acid **3** (green) compared to phosphate buffer (black); **(A)** Greigite; **(B)** Mackinawite; **(C)** Pyrrhotite; **(D)** Pyrite; **(E)** Magnetite; **(F)** Hematite. All experiments are done in phosphate buffer pH 7.0 in the presence of 10 mM hydroquinones and 50 mV/s scan rate.

As shown in Figure 3, the electrodes made of pyrrhotite, mackinawite and synthetic greigite reacted with water (black lines, all graphs). Greigite electrodes showed reversible redox process with an oxidation anodic peak at −190 mV and the corresponding reduction cathodic wave peak at −390 mV. The reversible nature of this process indicates that the electrode material was not dissolved during the scan. The peaks correspond to the reversible Fe^2+^/Fe^3+^ oxidation on the surface of the electrode (Benning et al., 2000). The pyrrhotite electrode showed two large cathodic waves at −160 and −385 mV, potentially corresponding to stepwise dissolution of polysulfur chains to sulfite ions with partial re-incorporation of the sulfur to the electrode surface during the anodic scan at −101 and 118 mV (Mikhlin, 2000). In contrast, pyrite and the metal oxides hematite and magnetite were stable under these experimental conditions.

### Electrode interactions with quinones

With the electrochemistry of the electrodes defined, the reduced quinones (**1–3**) were included in the measurements. While reversible oxidation and reduction waves were expected, Figure 3 shows that only pyrite, and partially magnetite, reacted with all quinones, and there was no detectable current change on the surfaces of more reduced iron sulfides. While there was no change in electrocatalytic behavior of mackinawite with methoxyquinone **2**, the presence of more oxidizing species **1** and **3** lead to dramatic drop of electrical current over all potential range, probably due to chemical oxidation of the exposed surface. Small increases in both cathodic (reducing, negative) and anodic (oxidizing, positive) currents were detected on the surface of the hematite electrode, however the current never reached a maximum value, indicating that at the applied potential values, the equilibrium was shifted toward reduced hydroquinones.

### Pyrite and magnetite electrodes

The mechanisms of redox reactions on pyrite and magnetite surfaces were then studied in more detail. As shown in Table 1, the electrocatalytic properties of pyrite and magnetite electrodes were tested in hydroquinone solutions of 10 mM concentration in phosphate buffer under nitrogen. The pyrite electrodes showed lower potential values of oxidation waves and higher (more positive) reduction waves for all quinones compared to magnetite, and, surprisingly, performed better than standard commercially available glassy carbon electrode. This indicates that the reaction of quinone on pyrite is more thermodynamically favorable than on other minerals. The measured value of the peak electric current (see Table 1) is the number of electrons flowing through electrode surface per unit of time. The current is proportional to the area of the electrode and the equilibrium constant of oxidation-reduction reaction on the electrode surface. Both mineral electrodes were composed of small particles of different sizes that contributed to roughness of the electrode, but this parameter does not significantly affect current and potential measurements (Menshykau et al., 2008). Areas of pyrite and magnetite mineral electrodes can be approximated to the geometric area covered by mineral particles, and currents registered at both electrodes under identical experimental conditions can be directly compared. Cathodic and anodic currents on pyrite electrodes were 10 to 30 times higher than on magnetite and more than 100 times higher than on hematite electrodes, suggesting that quinones were oxidized and reduced faster on pyrite than on other minerals.

**Table 1:** Summary of hydroquinone redox reactions on different mineral surfaces.

**Electrode**	**Hydroquinone**		**Methoxyhydroquinone**		**Naphthoquinone**	
	**E_a_(mV)/ i_a_(mA)**	**E_c_(mV)/ i_c_(mA)**	**E_a_(mV)/ i_a_(mA)**	**E_c_(mV)/ i_c_(mA)**	**E_a_(mV)/ i_a_(mA)**	**E_c_(mV)/ i_c_(mA)**
Pyrite	251	−3	284	−185	83	−83
	0.76	−0.48	1.12	−1.07	0.70	−0.75
Magnetite			119	−197	239	−137
			0.033	−0.045	0.07	−0.07
Glassy carbon	365	−89	306	−168	252	−145
	0.14	−0.088	0.22	−0.15	0.062	0.099

The difference between the peaks corresponding to oxidation and reduction was larger than the theoretical limit of 0.059/n expected for a fully reversible reaction. Larger separation between peaks seems to support a stepwise oxidation mechanism where hydroquinone is oxidized via one electron step to semiquinone, which is then rapidly oxidized to benzoquinone (Uchimiya and Stone, 2006). For pyrite, the ratio between the oxidation and reduction current peaks was 0.63 for **1**, 0.95 for **2** and 0.93 for **3**, instead of 1.0 as would be expected for fully reversible processes. On pyrite surfaces, reduction occurred at lower rates than oxidation for benzoquinone **1**, probably because of the formation of poorly soluble hydroquinone-benzoquinone complex that interfered with the reaction. In case of **2** and **3**, redox reactions were close to being reversible in both directions.

**Figure 4:**
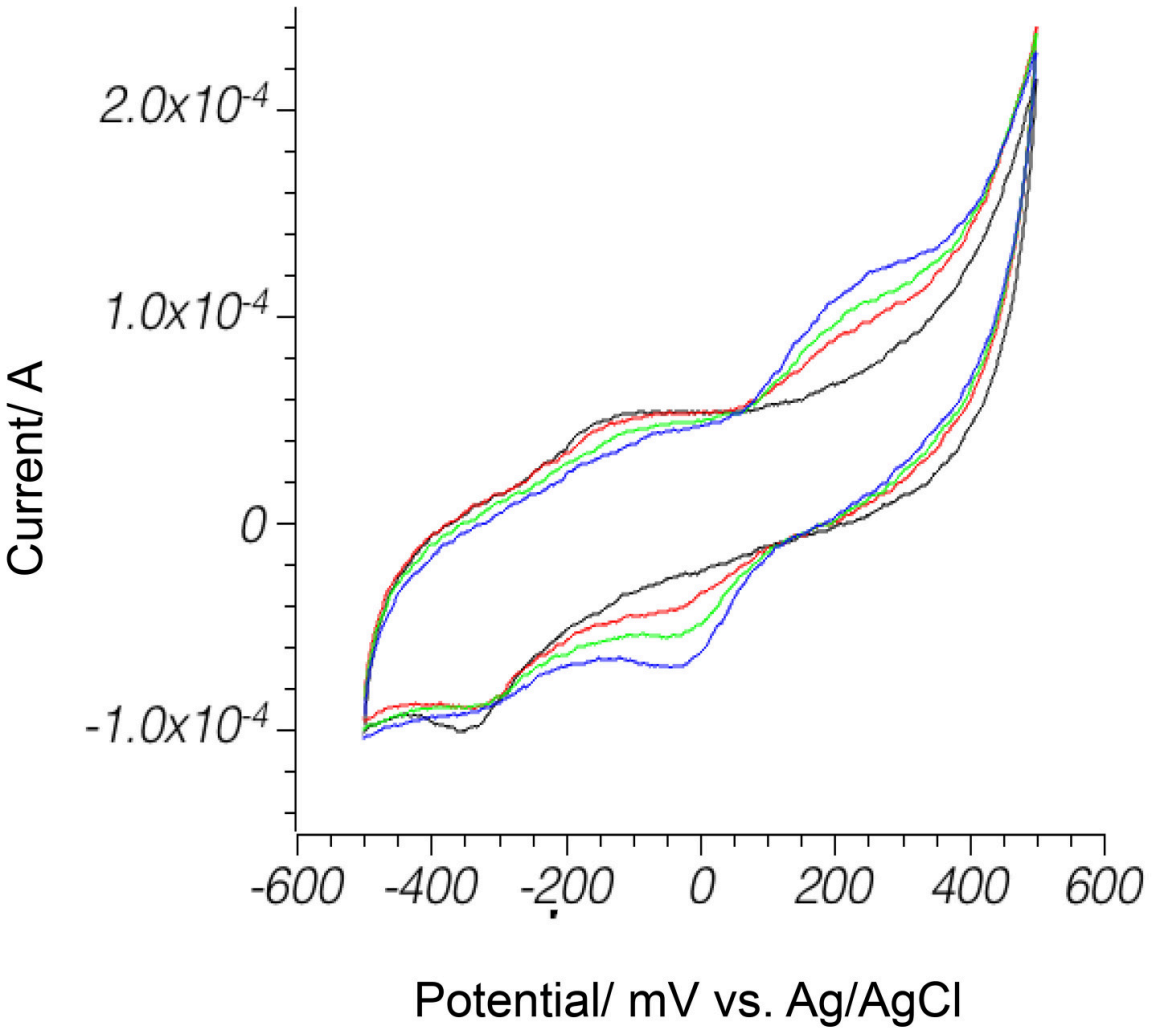
Hydroquinone **1** oxidation on pyrite electrode in anoxic environment. Phosphate buffer pH 7.0 , 50 mV/s scan rate. Black: bare pyrite; red: 0.2 mM H_2_Q; green: 0.4 mM H_2_Q; blue: 0.6 mM H_2_Q.

Concentrations of quinone used for initial screening were much higher than the naturally occurring range of these molecules, which is usually from 0.02 to 1 mM (Uchimiya and Stone, 2006; Marsili et al., 2008). Only pyrite showed an increase in current in the naturally occurring concentration range (Figure 4), and further studies were limited to electrochemistry of pyrite in the presence of 1 mM or less of hydroquinones.

### Mechanism of hydroquinones oxidation of pyrite electrode

**Figure 5:**
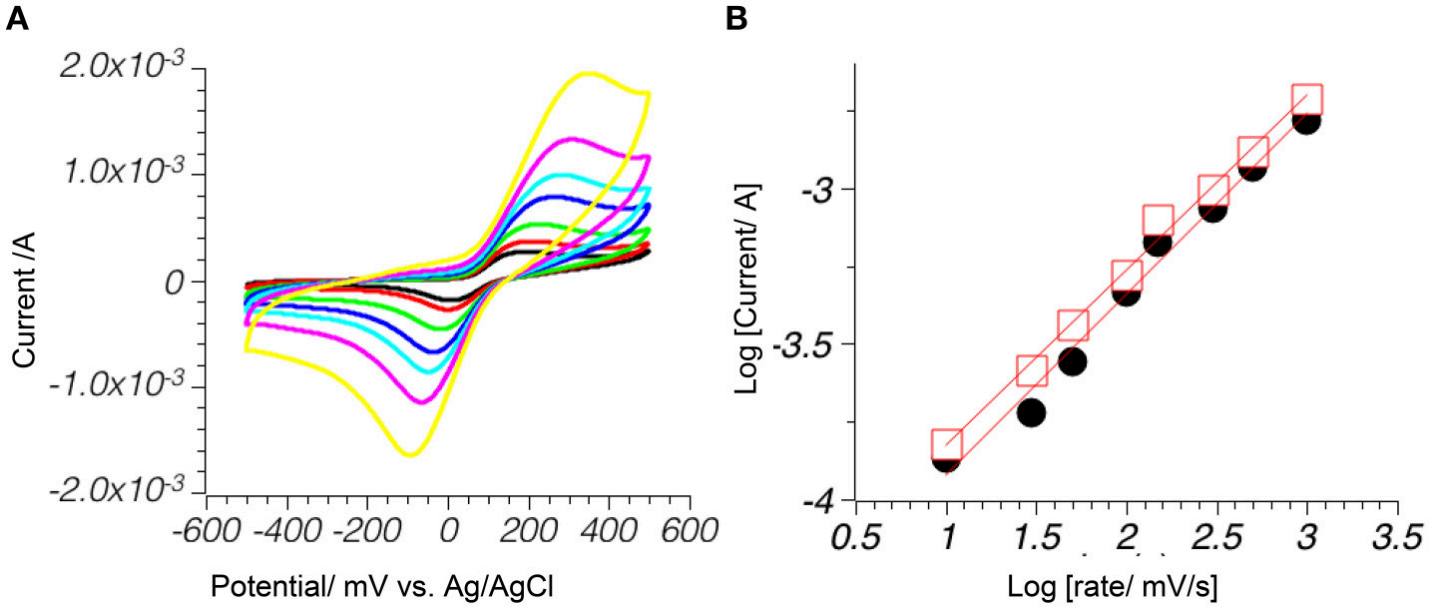
Current rise with the increase of the scan speed indicated a diffusion controlled mechanism. 1mM **1** phosphate buffer pH 7.0. **(A)** Successive scans with (from lowest to highest) 10, 50, 100, 150, 200, 300, 500, and 1,000 mV/s; **(B)** A log/log plot of change in peak current for absolute value of cathodic (black) and anodic (red) peaks vs. change in scan rate.

On the electrode surface molecules may be oxidized by diffusion or adsorption-controlled mechanisms. To distinguish between the two, the potential scan rate was gradually increased from 10 to 1,000 mV/s (Figure 5), and this change caused an increase in peak current. In diffusion-controlled processes, current rise is proportional to the square root of the scan speed, while in adsorption-controlled processes, the current rise is directly proportional to the speed of the scan (Batchelor-McAuley et al., 2010). A mixture of both mechanisms is possible in real systems. To distinguish between both processes, log|i_p_| (where |i_p_| is an absolute peak current value), was plotted against log(v) (where v is the scan rate in mV/s) (Gupta et al., 2012). The results showed a small contribution from the adsorption-controlled pathway in the case of unsubstituted hydroquinone **1** on pyrite surfaces, with the slope of 0.56 (*R*^2^ = 0.994) for the cathodic peak and 0.58 (*R*^2^ = 0.985) for the anodic peak, however slopes for **2** are 0.43 (*R*^2^ = 0.999) and 0.45 (*R*^2^ = 0.996) for pyrite electrode and 0.35 (*R*^2^ = 0.987) and 0.42 (*R*^2^ = 0.992) for **3** on magnetite electrode, showed that both processes were fully diffusion-controlled.

**Figure 6:**
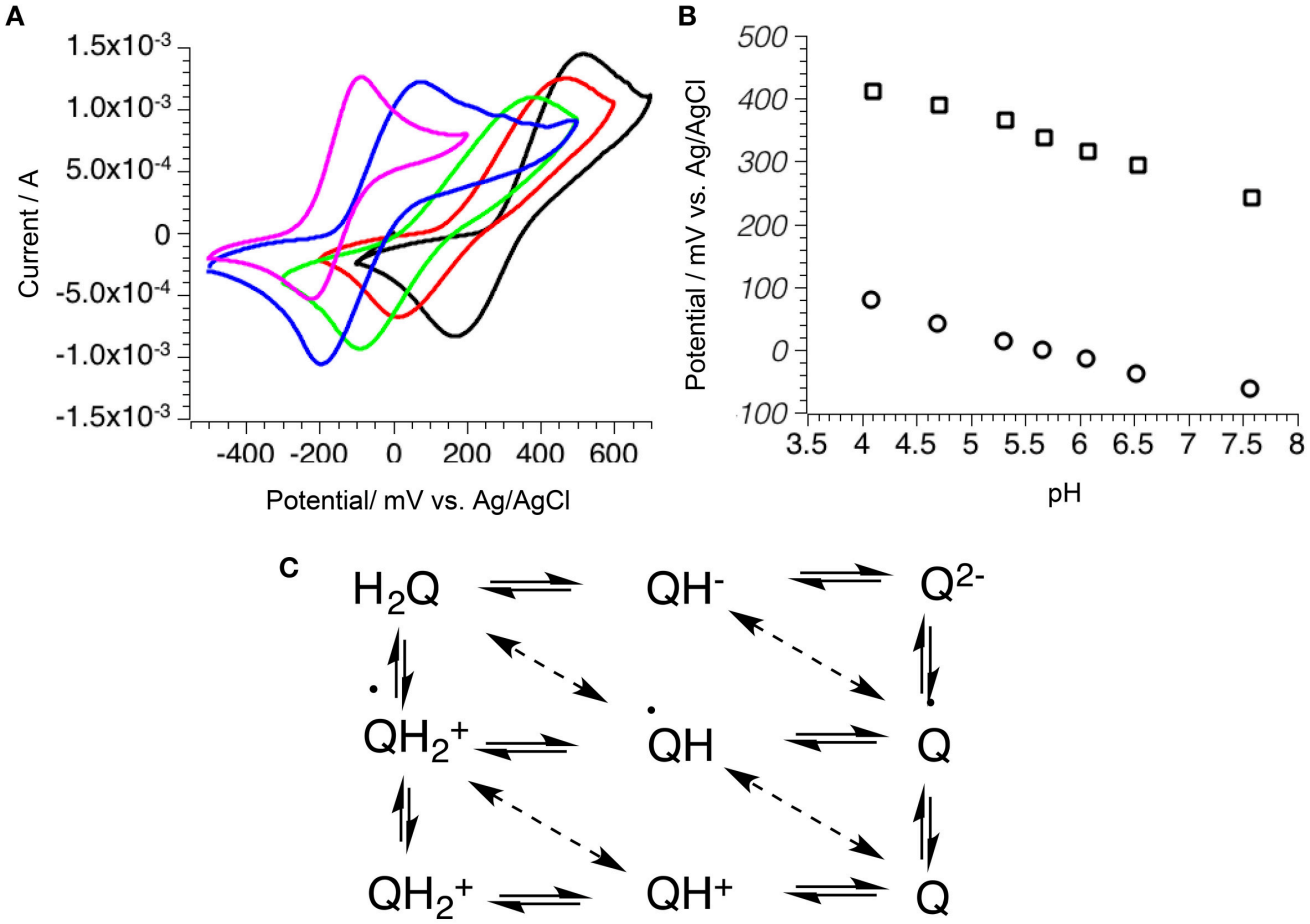
Mechanism of hydroquinone 1 oxidation of pyrite electrode as a function of pH. **(A)** Scan samples at different pH: 2.5 (black), 5.1 (red), 6.7 (green), 9.5 (blue), 11.8 (magenta); **(B)** Decrease of maximum potential peak with increase of pH for anodic (square) and cathodic (circle) peaks; **(C)** General diagram of quinone oxidation mechanism.

Variation of the redox potential with pH showed peak shifting to more reducing potentials with the increase of pH, indicating that both protons were involved in the redox process (Figure 6). Hydroquinone **1** has a pKa of 10.3, and below this pH the hydroquinone is mostly present in its diprotonated form (H_2_Q). The H_2_Q can be oxidized to benzoquinone (Q) or to semiquinone (Q^−^), which has pKa 4.3 and is deprotonated in the studied region (Quan et al., 2007) (Figure 1). The mechanism of the first oxidation involves two electrons and two protons, while the second is a one-electron two-proton process. The change in peak potential against pH was linear in the pH range 4 to 10 with the slope of −0.064 (*R*^2^ = 0.990) for anodic current and −0.058 (*R*^2^ = 0.995) for cathodic current. This value is close to −0.059, corresponding to a process where an equal number of protons and electrons are exchanged, suggesting that the process occurred via two-proton two-electron oxidation. The electrochemical oxidation of quinones on mineral surfaces has a different mechanism than quinone oxidations in solution, where hydroquinones are usually oxidized in two one-electron oxidations via a semiquinone radical intermediate.

### Electrode stability

**Figure 7:**
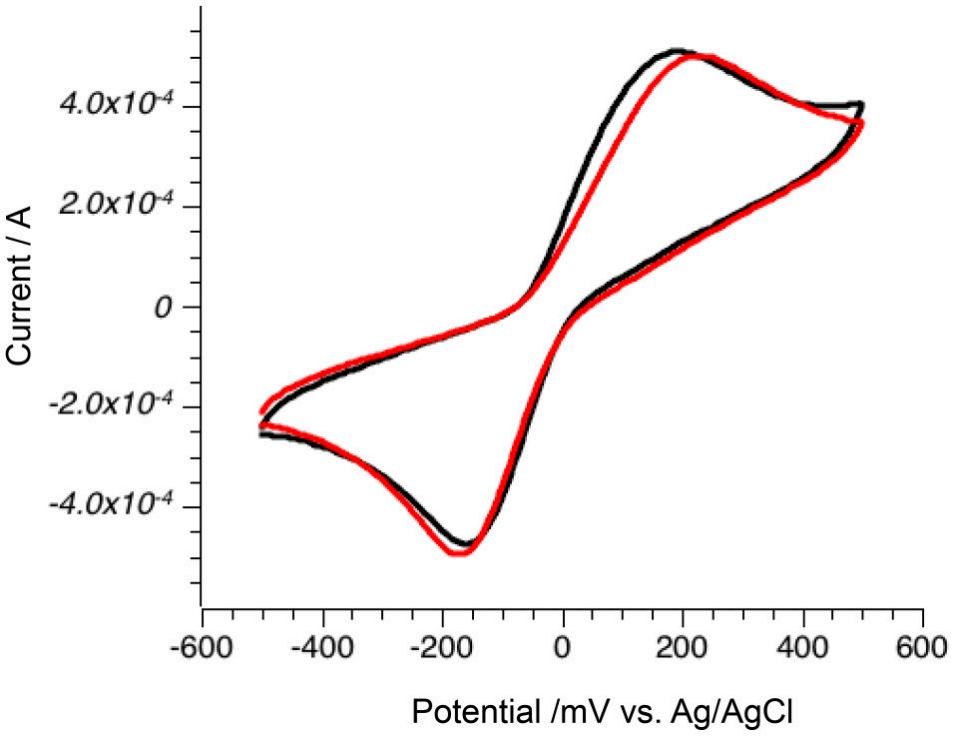
Stability of pyrite electrode. First oxidation scan (black) and 100th oxidation scan (red).

Stability of the electrodes under an argon atmosphere was tested over 100 cycles on pyrite electrodes with hydroquinone **2**. Small shifts from 200 to 210 mV for the oxidation peak and from −160 to −168 mV for the reduction peak were observed along with small increases (from −0.47 to −0.49 mA) in reduction current (Figure 7). Both observations are consistent with changes in electrode performance due to partial adsorption of the quinone to the surface, but the change is not significant and pyrite minerals potentially can exchange electrons with organic mediators over prolonged periods of time.

## Discussion

This work shows that long distance electron transfer can be coupled to abiotic organic redox reactions. To simulate processes occurring in the environments where electrochemical gradients are present, reactions on electrode surfaces made of common minerals were studied at the potential range of −500 to 500 V (vs. Ag/AgCl) expected to be found in natural environments (Baas Becking et al., 1960). Electrodes made of reduced metal sulfides are chemically unstable under the selected conditions and cannot transfer electrons to model organic molecules hydroquinones. This result is surprising: Reduced iron sulfides are usually considered to be the major catalyst of biogeochemical processes in hydrothermal vents (e.g., White et al., 2015). Observed small rises of current on the surface of metal oxides hematite and magnetite are consistent with the observation of very small effect of quinones on chemical dissolution of Fe(III) oxides (O'Loughlin, 2008). In contrast with other studied minerals, pyrite showed a significant current rise in the presence of low concentrations of quinones (0.2–1 mM), within ranges of concentration of organic matter in natural waters (Uchimiya and Stone, 2009). Electrical currents on pyrite surfaces are at least an order of magnitude higher than the maximum oxidation and reduction peak currents on other minerals, and occur at lower redox potentials, suggesting that the electron transfer between quinones and pyrite is a very efficient process that can dominate electrochemistry in natural environments.

The redox stability of pyrite in the presence of quinones can result in long term quinone cycling driven by potential differences between oxic and anoxic zones. Given the ubiquity of both pyrite (Vokes, 1993) and quinones (Scott et al., 1998), the out-of-equilibrium system generated by their interactions may have multiple impacts on the environment.

**Figure 8:**
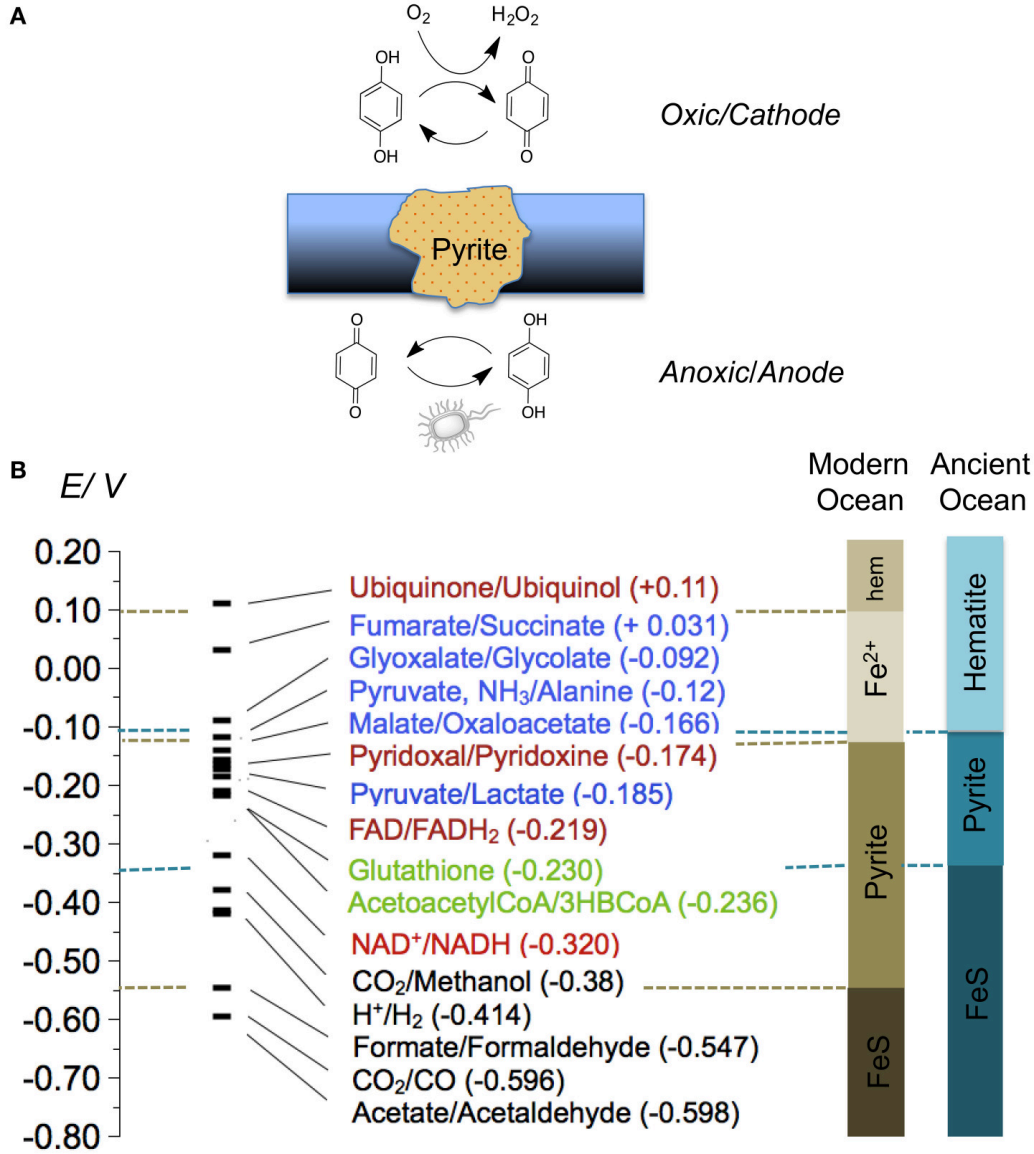
**(A)** Quinone cycling maintained by pyrite in the presence of a redox gradient; above: reduction of the quinone at the cathodic surface of pyrite leads to increased quinone cycling, ROS production, and oxygen consumption; below: hydroquinone reduction at the anodic surface of pyrite generates external electron acceptors for microbial respiration; **(B)** Redox potentials (25°C, pH 7.0, vs. SHE) of biochemical reactions relevant to the origin of life, compared to the electrochemical stability regions of iron minerals in modern and ancient seawater ([Fe]_T_ 0.5 × 10^−9^M, [S]_T_ 2.8 × 10^−2^M, and 2 x 10^−4^ M and 2 × 10^−7^ M, respectively); colors are used to indicate different biochemical roles of the molecules: redox cofactors (red), thiols and thioesters (green), primary metabolites (blue), and terminal electron acceptors (black); “hem” is hematite.

It has been shown recently that cable bacteria can form millimeter-long conductive filaments that couple sulfide oxidation in anoxic sediments with oxygen reduction in the oxygen-rich areas (Müller et al., 2016). Given that electrical current through conductive minerals can be coupled to hydroquinone oxidation, and that quinones are common external electron mediators used by a wide variety of bacteria (Hedrick and White, 1986), it is possible that other microorganisms apart from cable bacteria may also participate in biogeochemical processes involving long distance electron transfer. In this case our current models underestimate the role of abiotic electron transfer across the sediments (Seitaj et al., 2015) (Figure 8A).

Many naturally occurring hydroquinones are readily oxidized by dissolved oxygen to quinones. Continuous electrochemical reduction of the quinones back to the hydroquinones in the oxygen-rich areas may increase the ratio of quinone cycling and result in accelerated oxygen depletion of the solution (O'Brien, 1991) as well as abiotic generation of high local concentrations of hydrogen peroxide (Roginsky and Barsukova, 2000) (Figure 8A). It is possible that quinone cycling coupled to the long-range electron transfer is a complex reaction-diffusion system that can contribute to sharp seasonal oxygen variations.

Finally, hydrothermal vents and other naturally occurring zones where electrochemical gradients are found have long been considered as out-of-equilibrium systems relevant to the origins of life due to an apparent similarity between biological and geological electrochemical gradients (Martin and Russell, 2003; Amend and McCollom, 2009; Nitschke and Russell, 2009). In general the hydrothermal vent scenarios of the origins of life suggest that reduced metal sulfides were the precursors to the biological iron-sulfur redox cofactors and the catalysts of prebiotic reactions. The exact nature of the metal sulfide catalysts as well as the range of the relevant prebiotic reactions remains a matter of debate (Orgel, 2008; Wächtershäuser, 2016).

In Wachterhauser's “pyrite pulled metabolism,” a model proposed almost 30 years ago, pyrite served as a catalytic surface able to adsorb and concentrate negatively charged organic molecules, but reduced metal sulfides, such as pyrrhotite and amorphous FeS, were expected to participate in the redox reactions of the system (Wachterhauser, 1988). With few exceptions (Wang et al., 2012), reduced iron and niquel sulfides have been generally proposed as plausible minerals to catalyze prebiotic redox reactions instead of pyrite (Cody et al., 2000; Huber et al., 2012; Novikov and Copley, 2013).

Electrochemical systems have been recently suggested as a relevant approach to study prebiotic chemistry related to hydrothermal vents scenario (de Aldecoa et al., 2013; Barge et al., 2014, 2015; Herschy et al., 2014; Yamaguchi et al., 2014; Roldan et al., 2015). The focus of these studies is usually on abiotic CO_2_ reduction and reduced iron sulfides and sometimes iron oxides (green rust) are considered as possible electrode candidates. Focus on CO_2_ reduction as a main model system relevant to the origin of life has been chosen because of the common assumption that life started simply and evolved toward larger synthetic complexity (Martin et al., 2008). The opposite idea that life started from complex chemical mixtures (de Duve and Miller, 1991; Burton et al., 2012) is gaining more support with the growing realization that a large amount of organic material is formed abiotically in space and delivered to Earth with comets and meteorites (Chyba and Sagan, 1992). Under this scenario quinones, which have been produced abiotically under conditions simulating exoplanetary comet ices (Bernstein et al., 1999), are plausible first molecules relevant to the origin of life. The ubiquitous occurrence of many different quinones used by bacteria, fungi and plants suggests that quinones have a critical role in maintaining life on Earth (Szent-Gyorgyi and McLaughlin, 1983), but the role of quinones in early prebiotic reaction networks is presently underexplored.

The observed affinity between pyrite and quinones loosely resembles reactions in electron transport chain systems found across all domains of life, where redox and pH gradients across cell membranes are maintained, in part, by electron transport between metal sulfur clusters and quinone cofactors (Sun et al., 2013). Electron transport chain has a modular structure (Yankovskaya et al., 2003) and it is possible that a first primitive fragments of a biological electron transport chain appeared from abiotic electrocatalytic interactions between minerals and redox active molecules. In this case, an ability of pyrite surfaces to adsorb and concentrate amino acids and short peptides (Wachterhauser, 1988; Sanchez-Arenillas and Mateo-Marti, 2016) could have provided the link between the early redox systems and prebiotic reaction networks based on peptides and nucleic acids.

The redox processes of the living cells, with a few exceptions (Bar-Even, 2013), take place in the relatively narrow potential zone between −0.6 and 0.1 V (vs. SHE) (Weber, 2002; Bar-Even et al., 2012). If electrochemical reactions were important components of prebiotic redox systems, then minerals that acted as electrodes should have been stable in the potential window corresponding to prebiotic redox reactions, such as reactions involving redox cofactors, components of the citric acid cycle, thiols and thioesters. Side to side comparison of the redox potentials of these reactions to the stability of iron minerals at the same pH and temperature are shown on (Figure 8B). While CO_2_ reduction to organic molecules can take place on surfaces of FeS minerals, pyrite stability overlaps with a large part of biochemical redox potentials. Ubiquitous pyrite surfaces likely served as natural mineral electrodes in primordial reaction networks.

The simple system presented in this paper is a model that does not yet address many questions relevant to the natural settings. The selectivity and the stability of pyrite electrodes during the quinone redox cycling in presence of other organic and inorganic compounds will be studied in the next phase of the project. To understand the global impact of abiotic electrochemical reactions a model involving electrical currents across conductive sediments coupled with the diffusion processes at the sediment interphases has to be developed. Reduced iron sulfides are more electrically conductive than pyrite and magnetite (Telford et al., 1990), and are found at higher concentrations in the anoxic zones (Risgaard-Petersen et al., 2012). Possibly the electron flow across the sediments occurs mostly through the reduced iron sulfides, the electron transfer takes place at the pyrite interphase, and there is a competition between mineral corrosion and electrons transfer pathways. The complex pattern of the long-range electron transfer needs to be elucidated.

Present study of non-biological interactions between the organic molecules and the minerals suggests an existence of complex abiotic out-of-equilibrium systems driven by naturally occurring electrochemical gradients. These systems might contribute to global biogeochemical cycles and have an impact on different phenomena ranging from chemical pollution to the origin of life.

## Author contributions

OT designed and performed the experiments, analyzed the data, and wrote the paper.

### Conflict of interest statement

The author declares that the research was conducted in the absence of any commercial or financial relationships that could be construed as a potential conflict of interest.
