# Supplementary figures and images for: Electron Transfer between Electrically Conductive Minerals and Quinones
Source: Front Chem. 2017 Jul 13;5:49. doi: 10.3389/fchem.2017.00049 (PMC5508016; doi:10.3389/fchem.2017.00049)

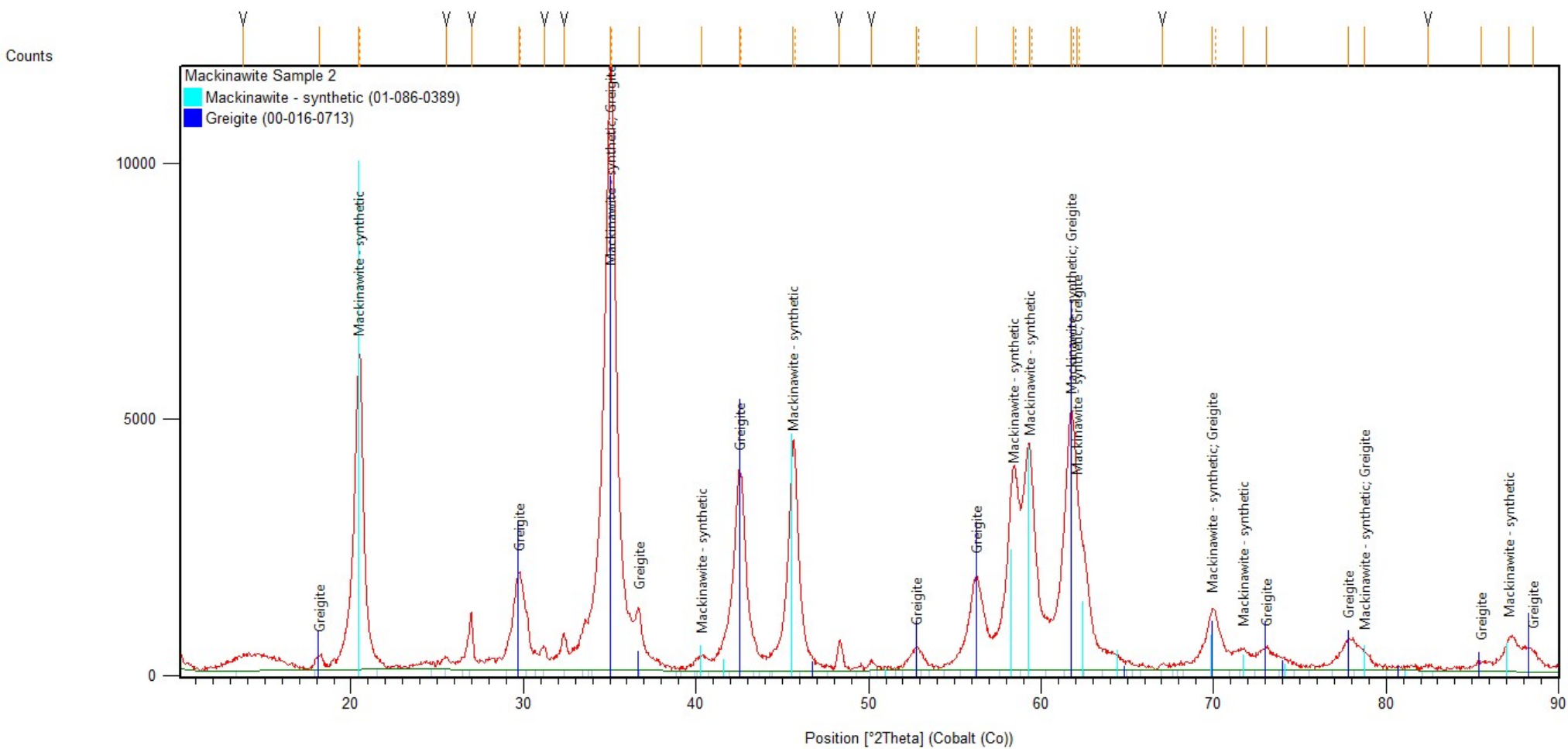

Supplement: Supplementary file 1 [file Image1.PDF]

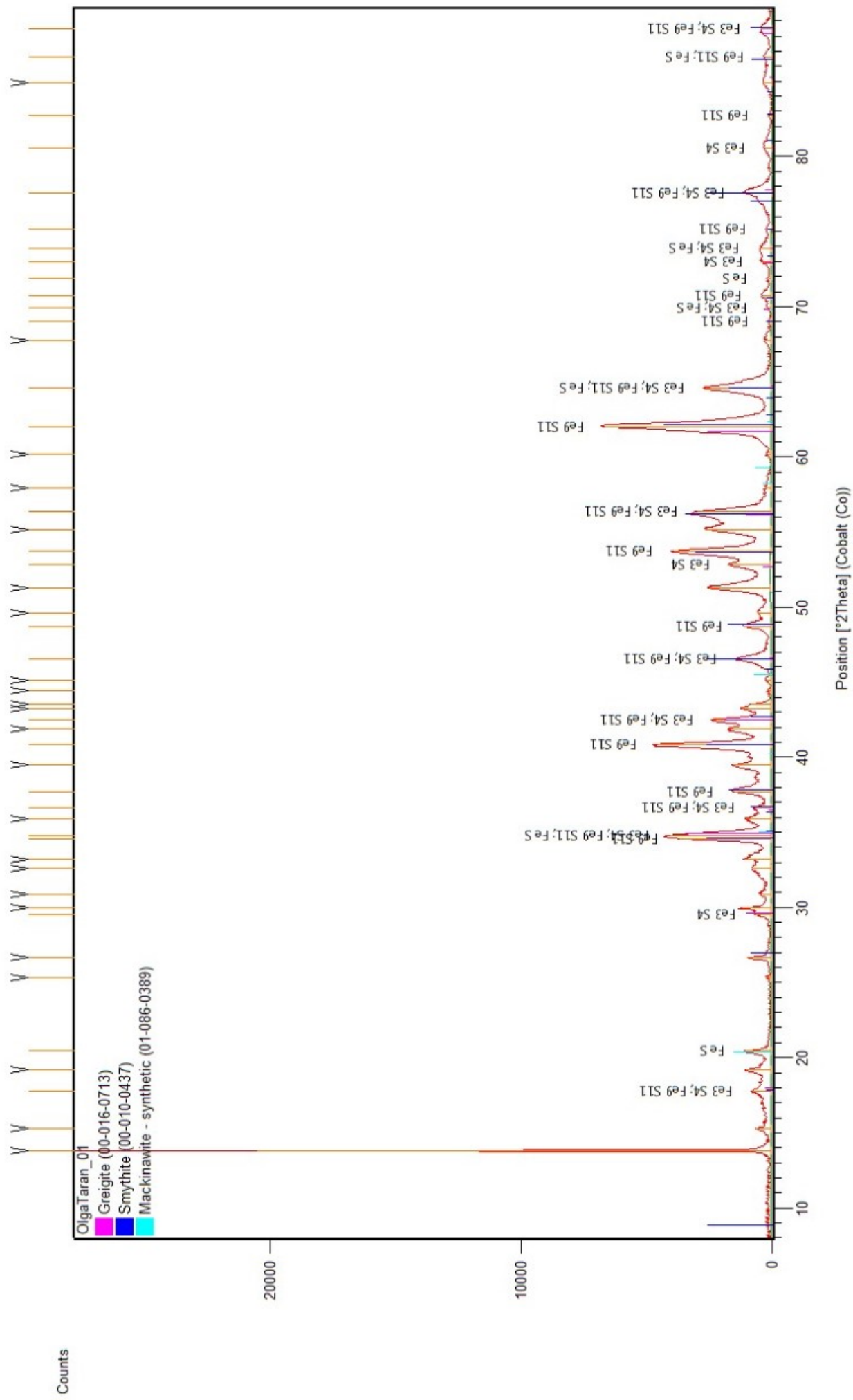

Supplement: Supplementary file 2 [file Image2.PDF]

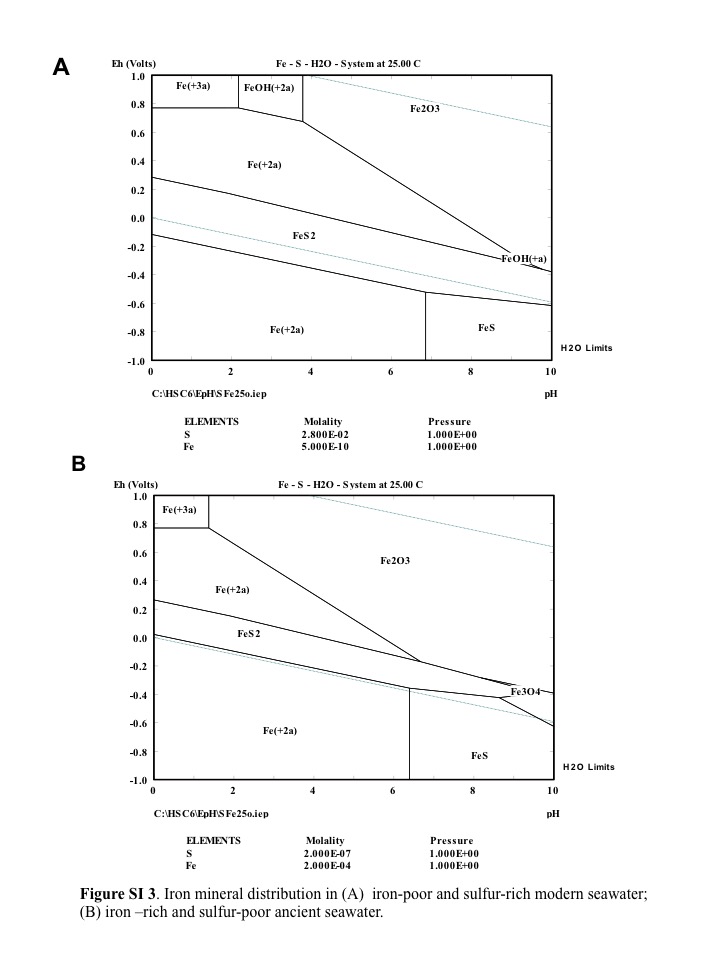

Supplement: Supplementary file 3 [file Image3.JPEG]
